# Supplementary material for: Consumption of Cooked Black Beans Stimulates a Cluster of Some Clostridia Class Bacteria Decreasing Inflammatory Response and Improving Insulin Sensitivity
Source: Nutrients. 2020 Apr 23;12(4):1182. doi: 10.3390/nu12041182 (PMC7230233; doi:10.3390/nu12041182)
Supplement: Supplementary file 1 [file nutrients-12-01182-s001.pdf]

Table S1. Dry cooked black beans composition.

Nutrient	g/100 g dry cooked black beans
Protein	17.82
Lipids	1.12
Dietary fiber	17.27
Carbohydrates	55.56
Resistant starch	3.49

*Results expressed in 100g dry basis. AOAC proximal chemical method. %humidity: 4.89%

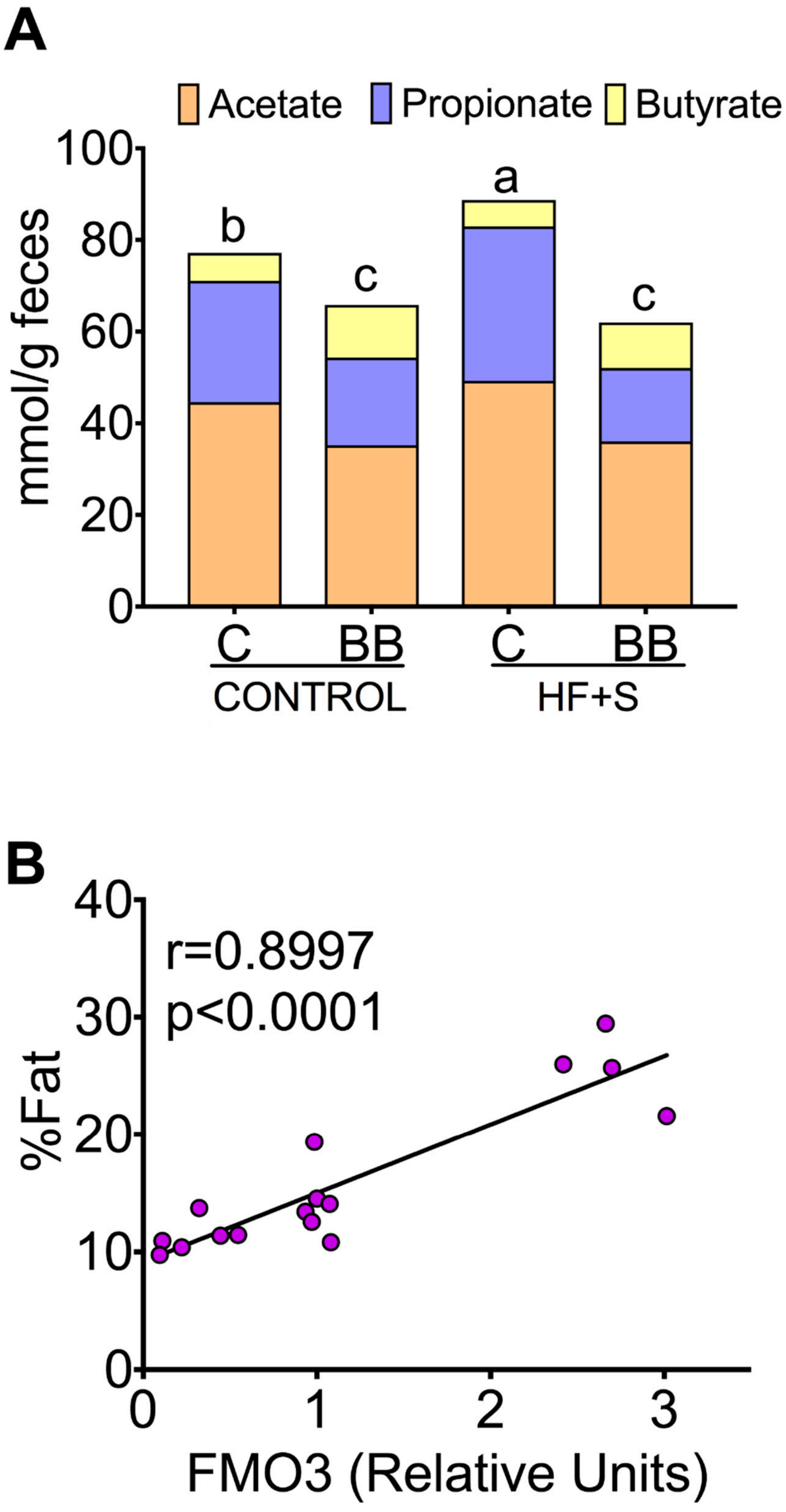

Figure S1. Total SCFA. (**A**) Fecal total short chain fatty acids, (**B**) Correlation between % Body fat mass and flavin monooxygenase-3 (FMO3).
